# Young men’s perceptions about the risks associated with sports betting: a critical qualitative inquiry

**DOI:** 10.1186/s12889-022-13164-2

**Published:** 2022-04-30

**Authors:** Christian Nyemcsok, Hannah Pitt, Peter Kremer, Samantha L. Thomas

**Affiliations:** 1 Institute for Health Transformation, School of Health and Social Development, Faculty of Health, Deakin University, Gheringhap St, Geelong, Australia; 2 Centre for Sport Research, School of Exercise & Nutrition Sciences, Faculty of Health, Deakin University, Gheringhap St, Geelong, Australia

**Keywords:** Young men, Sports betting, Risk, Public health

## Abstract

**Background:**

Gambling is an inherently risky activity. New technologies have led to the development of new, online forms of gambling such as sports betting, with round the clock availability and accessibility. While young men have been identified as a group that may be particularly vulnerable to the harms associated with these new online products, few studies have qualitatively explored young men’s perceptions of the risks associated with these products. Using concepts associated with the sociology of risk, this paper sought to explore the range of factors that may influence how 18–24 year old young Australian men conceptualise the risks associated with sports betting.

**Methods:**

Using a critical qualitative inquiry approach, in-depth interviews were conducted with sixteen participants in Victoria, Australia, who engaged in sports betting at least monthly. The data interpreted for this study included questions relating to awareness of gambling, the contexts associated with early gambling experiences; the factors that influenced current gambling behaviours, and why they engaged in gambling. A reflexive approach to thematic analysis was used to interpreted themes from the data.

**Results:**

Four key themes were constructed from the data relating to the factors that influenced risk perceptions. These included: 1) ‘The role of early experiences’, including exposure to gambling advertising in sport, and the gambling behaviours of social networks; 2) ‘The influence of peer rivalry and competition’, in which sports betting was used to form connections within and across peer groups; 3) ‘The normalisation of gambling’, including the embedding of gambling in everyday activities; and 4) ‘The influence of perceptions of knowledge, skill, and control’, including the belief that they could engage in responsible behaviours and stop gambling if they needed to.

**Conclusion:**

This study indicated that a range of factors may influence how young men conceptualise the risks and benefits associated with sports betting. Current public health strategies for gambling that focus on individual determinants and responsibility paradigms must be broadened to target the social and commercial factors that influence young men’s attitudes towards, and engagement in sports betting.

## Background

There has been increased recognition that gambling related harm is distributed unevenly and is a significant burden on the health and wellbeing of individuals who gamble, their social networks, and the broader community [1]. The commercial growth and diversification of gambling in the past decade had led to increasing calls from public health researchers and advocates for public health responses to prevent and reduce gambling related harm [2, 3]. Harms associated with gambling are unique to the individual, contributing to financial and interpersonal problems, as well as serious mental health illness and suicide [2, 4]. An evidence-based approach is important to understand the complex socio-cultural drivers of gambling engagement to develop robust public health interventions that prevent and reduce gambling related harm [5]. Researchers have identified that specific forms of gambling such as sports betting are associated with increased risk for gambling related harm [6]. While most studies have investigated a range of individual risk factors for harm associated with sports betting [7, 8], comparatively less research has qualitatively explored the range of socio-cultural and commercial factors for gambling that may influence risky patterns of engagement with sports betting.

Population-based surveys have indicated that young men are one demographic who may be at particular risk of harm associated with sports betting [9, 10]. A recent study found that young men were more at risk of the harms associated with sports betting products, because they were more likely to engage with these products, and place an increased number of bets as compared to women and older men [11]. Quantitative studies also indicate that there are a range of motivational factors for sports betting engagement that may increase young men’s risk for harm, such as to make money, to reduce boredom, and to demonstrate perceived knowledge of sport [7, 12]. Public health researchers have also qualitatively explored young men’s sports betting engagement and implications for risk and harm. Deans and colleagues [2016] conducted seminal research exploring the sports betting attitudes and behaviour of young men aged 20–35 years in Australia [13]. The researchers found that socio-cultural factors such as the embedded nature of sports betting in young men’s social and peer environments, and commercial factors relating to young men’s interaction with betting marketing, could increase young men’s risk for harm associated with their sports betting engagement [14, 15]. McGee [2020] investigated the sports betting experiences of young men aged 18–35 years and recommended that more effective public health strategies were needed to address the influence of betting marketing, as well as independent risk assessments for betting products [16].

Rhodes [17] describes that examining the relationship between health, risk perceptions, and behaviour requires distinguishing between whether risk is the product of individual decisions and actions, or if risk is embedded in social contexts and processes [17]. Rhodes explains that risk perceptions are significantly shaped by social interactions and norms, and argues that a more comprehensive understanding of risk that moves beyond the influence of individual processes, requires capturing the way in which risk is socially organised. This is important for improving the effectiveness of strategies to address the harms associated with unhealthy products, in which they better recognise the role of social processes in shaping risk perceptions and behaviour [18]. For example, researchers in alcohol harm prevention argue that exploring risk through social processes is important given that alcohol consumption is largely a social activity and is bound by socially shared meanings such as social connection [19, 20]. Furthermore, the researchers argued for the same approach to be extended to other unhealthy products that are influenced by social processes such as gambling [18]. Social risk theorists argue that examining the social processes that shape risk beliefs may help us to understand why people engage in activities that may be potentially risky for their health [17]. For example, Zinn [2019] explains that young people are motivated to engage in risk behaviours as part of developing their social identity and position in their social realm relative to their peers [21]. As such, social contexts are an important factor in understanding people’s motives for risk behaviour.

Individual motivations to engage in risky behaviours are complex and may be influenced by a range of everyday social contexts and experiences [17, 21]. These factors may influence whether a motive is driven by a desire to achieve material gain, or a response to a vulnerable situation [21]. For example, social contexts in people’s everyday environments may motivate them to make choices that are more favourable, or congruent with the choices of wider society [22]. Studies that have investigated young men’s sports betting attitudes and behaviours indicate that they perceive sports betting as a normalised practice for men who are engaged as ‘fans’ of sport, and this could also contribute to prompting their own betting participation [14, 16]. Another key dimension of risk that may explain why individuals engage in a risk behaviour relates to the control an individual perceives that they have over a behaviour [21]. Zinn [2019] describes that an individual with a high perception of control, may engage in risk to validate this self-perception, while an individual with a low perception of control may engage in risk to regain or enhance control [21]. Within these situations, social risk theorists explain that the degree of control is shaped through meanings that people interpret from their social environment [21]. This process involves reflexivity, whereby people attempt to makes sense of their control over a risk activity or behaviour through repeated interaction with their social environment [21, 23]. Put more simply, this means utilising the social environment to weigh up one’s perceived level of control over a risk behaviour. Research shows that social environments, and gambling products and marketing are key processes that influence beliefs about gambling risk and perceived control over gambling interaction [15, 24]. However, there is still limited qualitative research which explores how social and commercial factors shape young men’s perceptions of the risks and benefits of sports betting. Such research is important to broaden public health responses for gambling related harm to recognise the complex range of determinants that may influence how and why young men may engage in risky patterns of gambling.

Using concepts derived from theoretical considerations associated with the sociology of risk, this study sought to explore how socio-cultural and commercial factors associated with gambling shape young men’s sports betting risk perceptions and how risk behaviours are reflected in their sports betting engagement. Three research questions were explored:

1. What factors influence how young men conceptualise and reflect upon the risks associated sport betting?
2. Do perceptions of knowledge, skill, control contribute to young men’s motivations to gamble?
3. Do the findings highlight specific areas for intervention associated with young men’s sports betting and risk conceptualisations?

## Methods

### Approach

The data presented in this paper was part of a broader qualitative study that explored the gambling attitudes and behaviours of young men. A previous paper from this data explored the social practices associated with the young men’s gambling [25]. The study utilised a critical approach to qualitative inquiry. Charmaz [26] argues that critical inquiries:

… include concerns about social justice …. Addresses power inequality and injustice … (and is) embedded in a transformative paradigm that seeks to expose, oppose, and redress forms of oppression, inequality, and injustice [p. 35].

As Charmaz highlights, critical inquiries are grounded in the researcher’s value positions, and the positionalities that guide the development of the research. In terms of gambling, the researchers take a position that the risks associated with gambling products extend beyond individual behaviours and choices, and are influenced by a range of socio-cultural, environmental, and commercial determinants, including the marketing of gambling products. Critical inquiries are also consistent with public health prevention paradigms which state that the implementation of effective public health interventions should collect and synthesise evidence that:

… contextualizes the lived experiences of the individual gambler, within their wider social and cultural community, and alongside the commercial drivers and political contexts [27].

Constructivist Grounded Theory, which has strong pragmatist roots and complements critical inquiry through its commitment to social justice, guided the research process, including the study design and data interpretation [28]. This included a focus on co-constructing meaning with participants, and understanding how meanings and actions influence how they interpret different social phenomenon and the role of these phenomenon in their lives [29].

Ethical approval for the study was received from the University Human Research Ethics Committee (2019–402).

### Sampling and recruitment

The study sought to recruit young men aged 18–24 years from Victoria, Australia, who self-reported that they engaged regularly in sports betting (at least once a month). This age range was chosen based on evidence from gambling participation studies indicating higher levels of sports betting among young men aged 18–24 years when compared with other age demographics [9, 30]. Given that Australia legally permits gambling from age 18 years, the study provided an important opportunity to explore young men’s earliest legal interactions with sports betting and the range of factors that shaped their risk perceptions towards sports betting. Participants were referred to as ‘young men’ as this aligns with the age range for ‘young people’ (aged 16–24 years) as defined by the World Health Organisation (WHO) [31].

Convenience and snowball sampling strategies were used to invite participation to the study [32]. The study did not specifically aim to recruit young men who may have experienced harm associated with online wagering, however these sampling strategies helped to ensure that individuals with a range of gambling attitudes and experiences were able to participate in the study. As the interviews and data analysis progressed, purposive sampling was used to recruit participants who primarily engaged in sports betting, but also had experiences with other forms of gambling. These sampling strategies help to build depth in understanding participants’ gambling experiences. The researchers sought permission from a range of community organisations, including tertiary education providers, local councils and community sporting clubs, and gambling advocacy and support groups to distribute study information to their networks. Participants were also invited to share information about the study to their peers. Participants were informed that their participation was voluntary and required them to feel comfortable with sharing and discussing their experiences of sports betting. Participants who expressed interest were provided information about the study both verbally and in a written format and were required to consent prior to participation. Each participant received a $70 petrol voucher for their time, which was approved by Deakin University Ethics Committee, and was commensurate with tokens of appreciation given in other interview studies related to young men and gambling [13, 33, 34].

### Data collection

Semi-structured, telephone interviews were conducted with each individual participant between February and November 2020. The majority of interviews were conducted during the COVID-19 lockdown period in Victoria, Australia. Interviews lasted for a duration of up to 45 min and were recorded with participant’s permission.

Interviews collected socio-demographic information including age, postcode, employment status, occupation, relationship status, living arrangement, and highest level of schooling. After the first interview, The Problem Gambling Severity Index (PGSI) [35] was subsequently administered to each participant to provide a broad indication of risk of problem gambling. Interview questions were flexible to ensure that the data was co-created with participants. As the interviews progressed, and concurrent data analysis occurred, themes and lines of questioning were revisited. The open nature of questions and prompts, encouraged participants to reflect on their experiences with gambling, and elaborate on areas of discussion that were important to them [36]. This study focuses on questions that related to participant’s awareness of gambling and the contexts associated with their first gambling experiences; the factors that influenced their current gambling behaviours, and their rationale for engaging in gambling.

As Varpio et al. [2017] argue, data saturation in qualitative studies is a ‘thorny concept’ (p. 46) [37]. Braun and Clarke [38] state that decisions about when to stop data collection are subjective and situated in a range of meanings and contextual factors relating to the data that is collected. Considerations about the sample size were constantly reflected upon throughout the data interpretation process. We utilised guidance from Malterud et al. [2016] regarding sample size whereby we reflected upon the study aims, the specific nature of the sample, the theoretical concepts of the study, and the quality of the data (dialogue) with the participants [39]. A decision to stop collecting data was reached when the researchers determined that the interpretation provided enough ‘information power’ to address the study aims and research questions [39].

### Data analysis

Author one transcribed all the interviews, with sufficient space between the interviews to interpret and identify patterns in the data (which were explored in subsequent interviews). Data were interpreted using Braun and Clarke’s reflexive thematic analysis [40, 41]. While the overall study was guided by theoretical concepts associated with social norms, the concept of risk was reviewed in the literature and utilised as a theoretical concept that could underpin the data. The coding process was open and data-driven. Initial coding focused on semantic (or surface) meanings in the data, followed by a process of in which latent (or underlying) meanings were considered, and refining these to construct overarching themes and sub-themes. The researchers maintained regular contact throughout the analysis process to discuss and reflect upon interpretations and patterns in the data and how these could be explained by key concepts of risk as well as to plan for the written analysis. Data extracts were selected that conveyed participant’s meanings that were grounded in the data [42]. A model was then constructed to conceptualise young men’s risk perceptions and behaviours associated with sports betting.

## Results

The full details of participant’s socio-demographic and gambling characteristics have been presented elsewhere [25]. In brief, sixteen young men, aged 18–24 years (*M* = 20.81) participated in the study. The majority lived at home with a parent or guardian (*n* = 14). Most had completed high school education at Year 12 (the final year of Australian high school) (*n* = 10), just under a quarter completed Year 11 (*n* = 3), and just under a quarter completed education beyond high school (*n* = 3). Three quarters of participants were employed full time (*n* = 12). Scores on the PGSI indicated a range of gambling risk profiles among participants, including four ‘non-problem’ gambling, five ‘low risk’ gambling, three ‘moderate risk’ gambling, and four participants who indicated ‘problem gambling’.

Four themes relating to risk were constructed from the data. These themes related to the influence of early gambling experiences, the role of peer rivalry in sports betting risk perceptions, the normalisation of gambling in everyday activities, and the influence of knowledge, skill, and control.

### Early gambling experiences in shaping gambling risk perceptions

A range of experiences with gambling prior to the legal age of 18 years for gambling in Australia shaped participants’ early risk perceptions. Most participants had an awareness of sports betting during their adolescence through seeing gambling advertising when watching professional sport or through their social experiences that were associated with gambling. These social experiences included participating in informal gambling during major sporting events with family members and or peer networks, and being present when family members or peer networks engaged in gambling. While a few participants also engaged with gambling products such as ‘scratchies’ (instant lottery cards – also known as ‘scratchcards’). Scratchies were bought as a gift by a parent or relative and participants recalled that they did not perceive scratchcards to be a type of formal gambling. Participants discussed multiple social experiences with gambling during their adolescence:

“*The Melbourne Cup* (horse race) *was the only time I ever bet on a horse when I was young. With scratchies, since about 10, I had my parents buy them for me and then scratch them and then win something. I have an older brother and he would go to the Returned and Services League of Australia (RSL) and bet with all his mates’*” – Participant nine, 19 years old, problem gambling.

Some participants’ early risk perceptions about their capability with gambling appeared to be shaped through activities not related to gambling. This involved competing with peers. These activities related to making predictions about the outcome between two sporting teams, colloquially referred to as sports tipping, or creating virtual sporting teams based on the best performing sporting players, known as fantasy sporting leagues^1^. Participants stated that the competitive nature of these activities helped to enhance their engagement as a ‘fan’ of sport, and peer connections. In terms of risk, engaging in these activities created a sense of knowledge and understanding about sport and how to predict the outcomes of sporting matches. This contributed to shaping a perception of control relating to their future sports betting attitudes and behaviours. For example, the following participant recalled that his interest in sports betting was shaped through his prior engagement with fantasy sporting leagues.

“*I was big into the fantasy hockey, fantasy soccer, fantasy basketball, fantasy football. I guess technically we used to put money on for these fantasy one’s as well. I guess it helped me increase the knowledge of some of the players and teams. It also helped enjoy watching them too, a bit more invested*.” – Participant sixteen, 24 years old, moderate risk gambling.

Furthermore, some participants developed reduced risk perceptions towards gambling through learning how to weigh up the risks of winning and losing. Reduced risk perceptions were shaped through modelling the individuals in their social networks who regularly engaged in gambling, including their male family members and peers. For example, they learned about the technical aspects of sports betting through members of their social networks such as the meaning of specific odds and betting markets as well as how to place a bet. This was particularly captured in one participant’s account of his early experiences:

“*Well my stepdad, when I was younger he would explain to me that if it’s paying $1.50, you’re gonna* (going to) *get this back for it, you know, one of them with better odds and they’re most likely to win. You’d ask –* ‘*what’s a quinella or an exacta*’ - *and he’d give that to me and yeah, I guess I had like half an idea of what was going on*.” – Participant ten, 19 years old, moderate risk gambling.

### The role of peer rivalry in sports betting risk perceptions

Peer group rivalry played a role in participant’s risk perceptions towards sports betting. This was particularly highlighted by participants who scored a ‘no’, ‘low’ or ‘moderate’ risk gambling. This routine involved competing against peer group members through betting. For some, peer group rivalry within social groups was shaped prior to formal and legal engagement in sports betting. For example, a few participants mentioned that they participated in informal gambling when they engaged in playing a team sport with their peers or when watching live sporting matches and predicting a winner:

“*While we were all under 18 we might have little bets with each other – like I bet you my team is going to beat your team and the winner has to buy lunch or something – but nothing official on gambling websites*.” – Participant fifteen, 24 years old, non-problem gambling.

Rivalry through sports betting enhanced peer connections, and this influenced some participants to be less inclined to consider risk in the context of their betting. For example, the following quote illustrates how enthusiasm associated with peer group rivalry through sports betting was embraced and meant that there was less reflection on potential risks:

“*It’s always good with your mates because if you watch a game with your mates and all your mates would have different bets on, so you’re all like fuckin going at each other, trying to win a bit. It becomes like a competition*.” – Participant four, 18 years old, low risk gambling.

When asked to reflect on the impact of engaging in rivalry on their betting engagement, a few participants acknowledged that there were risks associated with this practice. For example, the following participant reflected that engaging in rivalry contributed to increased frequency of both his own and their peers’ betting.

“*Definitely be an increase in betting overall because we’re discussing it with each other. It would be some friendly competition between us and that way it would be a way for us to bond in general, but yeah, always increase our betting talking about it*.” – Participant fourteen, 22 years old, moderate risk gambling.

### The normalisation of gambling in everyday activities in reducing perceptions of risk

There were regular social cues to gamble that were embedded in participant’s everyday lives, which alerted their attention to sports betting and in turn nudged their betting intentions and engagement. This was mentioned by participants regardless of gambling risk on the PGSI. The most typical social contexts were recreation, work, and educational environments. These social contexts had implications for risk because participants attached positive meanings that sports betting was more readily accepted and permitted within these settings. The following participant described in detail that there were multiple and regular cues in the social environments of young men that prompted their betting intentions and engagement:

“*I think gambling is just always around what young men are doing. Like most young guys love sports and so even if you’re not into gambling because you’re around sports, you always hear about it, you see about it. You’re a young guy so you probably go out to the pub to drink and see gambling or you go to the city and you see the casinos. I think it’s just the exposure to it, how gambling is seen a lot in young men’s life*.” – Participant four, 18 years old, non-problem gambling.

Sports betting was also normalised within everyday recreational settings that were culturally and socially valued by the participants. For example, betting was intrinsically linked as part of a range of activities that were associated with watching live sport with friends. Furthermore, betting sometimes took place alongside other risky activities such as the consumption of alcohol. When some participants were asked to reflect on how alcohol consumption could influence their sports betting engagement, a few were guarded about the associated risks:

“*Not necessarily, you didn’t want to go stupid if you got a bit too drunk, you didn’t want to put too much more on a horse. So if you got too drunk and put like 100 bucks on something, that’s silly, so no, I wouldn’t say it impacted too much*.” – Participant two, 20 years old, low risk gambling.

Another participant acknowledged that the combination of these activities could influence risky patterns of sports betting and described that it required him to implement some limits on his spending to reduce monetary risks when consuming alcohol in social settings and engaging in betting:

“*I’ve got the limit, but if your mate’s drunk as well then you don’t care (laughs). So sometimes you end up getting a bit more money out. But the way I normally do it is I move a certain amount of money into my spending account and I lock off my bank account until the next morning. Then I can just look at my cash and be like – ‘this is how much I have*’*.*” – Participant four, 18 years old, non-problem gambling.

Some participants, in particular those who scored as ‘moderate’ risk or ‘problem’ gambling on the PGSI, indicated that their participation in betting was impacted by aspects such as conversations about betting and engagement in betting by peers. These aspects were described as difficult to escape due to their presence in multiple social settings including recreation settings, as well as their workplace and educational settings. This suggested that the boundaries of betting being a social activity were removed because it became part of settings that were not recreational spaces tied to watching sport. As a result, it contributed to risks associated with gambling being amplified. For example, one participant stated that due to the high number of his peers that held a betting account, conversations about sports betting were “*never ending*”. Another stated that the regular occurrence of sports betting at his workplace during lunch breaks prompted his own betting, and this influenced him to engage in more frequent betting within this social context:

“*Just at lunch times really, all the guys at work were betting so I kind of just joined in because they’re doing it. Because sometimes I work by myself and sometimes I work with others, and when I’m with the others I definitely bet more during the day because they’re big on the horses*.” – Participant thirteen, 21 years old, problem gambling.

There were also direct social cues that prompted engagement in betting. When together, young men showed each other information about betting on their phones. However, social messaging apps (SMA), such as WhatsApp and Messenger, enabled them to continue discussions about betting even when they were not together. Some participants described instances in which SMA nudged their own engagement with betting. For example, live sports often prompted participants to have discussions about betting on SMA and this in turn contributed to subsequent engagement in betting. Risk perceptions appeared to be reduced because the prompting of betting encouraged participants and their peers to feel united when they were betting. The following participant described that conversations about betting on SMA were frequent in his peer group and this contributed to his experience of risky betting:

“*Whenever the footy is on, we’ve got our group chat and we send our screenshots of our multi’s* (betting accumulator) *or we’ll do a joint multi and talk about it*. *I’d see all the screenshots of everyone’s multi’s and then I’d be like –* ‘*they’re wasting their money*’ *– and then all of a sudden I’ve sent like 3 or 4 in a row and I’m like –* ‘*shit*’ *– and then you look at it and you’ve just wasted money*” – Participant ten, 19 years old, moderate risk gambling.

Furthermore, a few others identified that these regular nudges, and the social nature of SMA groups that facilitated these nudges, created risks because it prompted them to place bets when they were trying not to engage in gambling. For example, one participant described that group chats about betting made it difficult for him to avoid thinking about gambling even when he had tried to reduce his engagement:

“*I’ve tried to really back off of late, but it’s hard. Just because you put yourself in position with friends that do it, and then you’re in group chats and someone posts something and then that sort of triggers stuff, you’re thinking about it again, and then you’re like – ‘I thought I was trying to not gamble’ – and then you start gambling*.” – Participant five, 22 years old, moderate risk gambling.

### The influence of knowledge, skill and control in risk perceptions about betting

Participants, regardless of PGSI score, actively engaged in reflexivity through weighing up the risks associated with sports betting in comparison to other forms of gambling. This in turn had important implications for control over their sports betting. For example, there was a common sentiment that outcomes tied with betting were less random and less associated with chance in comparison to electronic gaming machines and casino gambling, and sporting knowledge – particularly relating to sports they enjoyed and supported—played a key role in betting success and financial rewards:

“*I personally don’t like casinos or the pokies because there’s no knowledge that can help you win. I like to think that my knowledge can help put the odds bit more in my favour – whereas if you’re spinning at a roulette table there’s nothing really you can do to control that*.” – Participant three, 21 years old, low risk gambling.

Perceptions of control were evident in participant’s accounts of a range of strategies that they described could be used to reduce the risks associated with sports betting and enabled a competitive edge in winning money. They stated that these strategies could positively influence the outcomes of their bets, and also influenced the types of betting products and markets they favoured, with particular engagement in high-risk markets. As an example, many participants described engaging with ‘same game multi’ markets; a product that enabled the accumulation of multiple selections on different outcomes within one sporting event. They indicated that bets through this type of product could result in more immediate rewards. This, as well as the association between having a significant interest in sport and a perception that they had a high level of sporting knowledge, influenced the appeal of multi bets. This perception of control led a number of participants to perceive that engaging with this type of product reduced the risks associated with losing, describing these bets as “*low risk, high reward*”. However, as the following quote illustrates, these markets, alongside risk reducing promotions such as inducements and cash back offers, contributed to a few participants staking more money on these markets:

“*Same game multi’s for me. There’s a $50 cash back if one leg of your same game multi fails, so I do same game multi’s when that promotion is on. I wouldn’t do anything else. I tend to put more money on a same game multi if there’s a promotion like that, so, I would say I’d put on an obvious multi, I would put 50 bucks instead of if the promotion wasn’t there I’d probably put 20 bucks*.” – Participant seven, 19 years old, problem gambling.

Although participants indicated through their experiences that they engaged in sports betting regularly, some believed that they had control over their gambling in comparison to other young men in their social circle because they engaged in wagering in a way that reduced their risk. For example, a few of these participants stated that their sports betting was comparatively less frequent to their peers:

“*I think there’s definitely a lot of guys out there that definitely bet a lot more than me*.” – Participant thirteen, 21 years old, problem gambling.

Others claimed that they knew when to stop gambling in comparison to their peers:

“*I find it pretty easy to unattract myself, I find that I’m much better than people I know*. *I have one mate that is easy on spending $100. There’s just no switch on them*” – Participant nine, 19 years old, moderate risk gambling.

A few perceived that they were more careful in the money they spent on betting:

“*The system I have in place, I’m not betting stupid amounts like I know my mates do*. *I’m not too heavily invested in it. I’ve got one mate who bets pretty large amounts and uses it as a way of making money*” – Participant one, 19 years old, PGSI not measured.

Participants who indicated that they had control over their finances and ability to limit the amount of money that they spent on gambling had a decreased perception of the risks associated with products. Most participants indicated that they allocated themselves a spend limit for their betting, with some specifically setting a fixed deposit limit within their betting app. However, some participants described situations in which they perceived that their fixed deposit limit was financially restrictive for their betting and subsequently increased this limit to allow for more regular betting or an increase in spend. Again, perception of control was exemplified by a few participants when rationalising this decision:

“*I had one* (deposit limit) *at the start but I bumped it up because I’m pretty good with my self-control*.” - Participant sixteen, 24 years old, moderate risk gambling.

Finally, was the role of marketing and promotions in creating perceptions of knowledge, control, and reduced risk perceptions. Participants engaged with marketing and promotions through viewing a range of information about different betting products and markets, and promotional offers and inducements that encouraged engagement with betting markets. This shaped some participant’s perceptions that being smart or strategic with promotions could contribute to gambling success. For example, some described actively looking for, and *“taking advantage”* of the best promotions and price discrepancies between companies, with one participant discussing that this was part of developing skill and knowledge about betting:

“… *if you’re going to be smart with it or if you do it at all* (betting), *you’d want to get the best value for money*.” – Participant two, 20 years old, low risk gambling.

Direct to consumer marketing encompassed betting promotions and inducements that participants perceived reduced the risks associated with betting (and losing their money). Participants regularly engaged with or were prompted by sports betting companies to view this content. Direct marketing came in a range of forms, including emails, text messages, push notifications, phone calls, in-app promotions, and social media marketing. A number particularly mentioned receiving matched deposit offers, in which a total deposited sum of money was matched as a bonus bet. The perceived benefits tended to outweigh considerations regarding the risks associated with taking up these offers:

“*That’s where I made my first big deposits, because at the start I was probably depositing $5-10 a month or something. But then they would offer matched deposits that if you deposit $300, they’ll match all $300.*” – Participant twelve, 23 years old, moderate risk gambling.

However, a number of participants critically reflected on the risks associated with these promotions. For example, a few recognised that these were strategies that sought to encourage gambling. Others were concerned that these promotions would contribute to them engaging in more regular and risky patterns of gambling. While some talked about turning off notifications from their betting accounts, one participant talked about the fear of missing out on good promotional offers and deals:

“*It gets annoying and most aren’t relevant. But I also don’t want to turn them off in case they offer something that might appeal to me.*” – Participant eleven, 20 years old, low risk gambling.

## Discussion

**Fig. 1:**
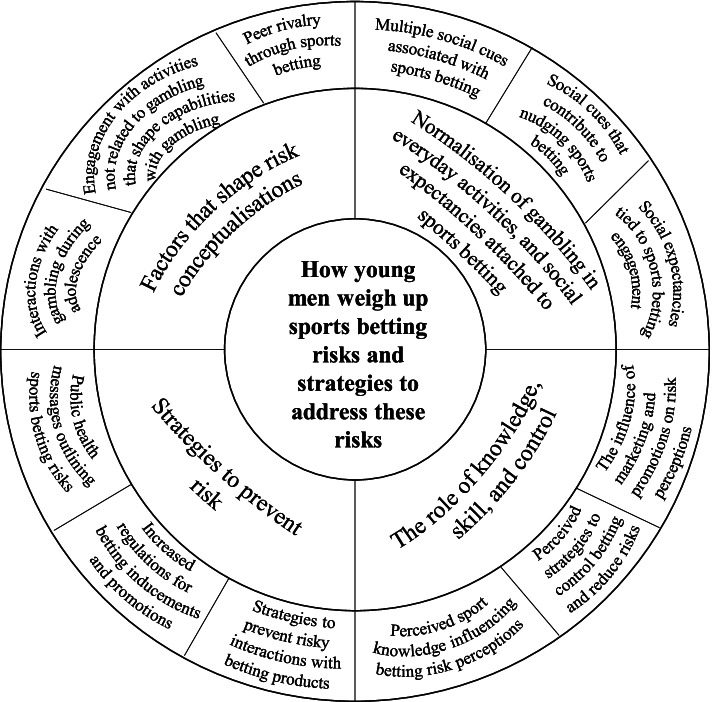
Understanding how young men weigh up the risks associated with sports betting, and strategies to prevent these risks

This study aimed to provide detailed information about how perceptions of risk are shaped by a range of individual circumstances, early experiences with gambling, social contexts and pressures, and industry practices. These insights are important given the range of public health and health promotion interventions for this population sub-group appear to focus specifically on personal responsibility behaviours. Figure 1 depicts a model showing descriptive examples associated with four key themes relating to how young men weigh up the risks associated with sports betting, and how these risks could be prevented. The four themes relate to: the factors that shape how young men conceptualise risk associated with gambling; the normalisation of gambling in young men’s everyday activities, and the social expectancies tied to sports betting; the role of knowledge, skill and control is risk perceptions; and key strategies that may prevent these risks. The findings raise three points for discussion.

First, the study showed that participant's conceptualisations of risk associated with sports betting were shaped by a range of social and commercial interactions with gambling and non-gambling activities during their adolescence. This aligns with a significant body of research conducted with young people prior to the legal age for gambling, that has highlighted how social contexts for gambling and commercial factors such as gambling marketing, influence young people’s beliefs about risk and future gambling intentions [43–45]. As such, participants in this study may have developed attitudes towards sports betting through these interactions during their adolescence that were influential in the uptake of sport betting. This may be explained by the contention of social risk theorists who argue that risk perceptions are shaped and modified by social contexts and processes [17, 21]. The implication here is that prior to the legal age for gambling and as part of a comprehensive public health approach, public health messaging could be targeted towards informing young men about the risks associated with gambling, in turn countering the developing positive or normalised perceptions towards sports betting that may increase their risk for harm.

Second, the study found that there was a link between a range of social cues and social interactions associated with sports betting, and participant’s sports betting motives and risk conceptualisations. Social theorists explain that the social environments people live within shape their understanding of risk as well as the acceptability of taking risks [17, 21]. This means that regular social cues for sports betting in young men’s environments may signify to them that betting is a normalised and accepted activity. Furthermore, people may also engage in risk behaviours as part of their identity formation and engage in risk activities to evaluate or protect their social position or status [21]. This suggests that a determinant of young men’s motives for sports betting as a risk activity, may relate to maintaining their position within social and peer interactions. Public health researchers who focus on alcohol consumption, explain that there are limitations to public health measures that focus solely on risky alcohol consumption because drinking is not necessarily the defining feature within the social interactions of young people [46]. This lesson from alcohol control suggests that public health responses for gambling could target the influence of social contexts in young men’s betting motives. Given that individuals may experience serious harms from gambling [2, 4], implementing this public health measure may help prevent harm among young men.

Third, the findings from this study indicated that knowledge, skill, and control reduced beliefs about risks and contributed to risky patterns of sports betting regardless of their individual risk or problem gambling status. This in part may be explained by the way perception of control is considerably dependent on the social conditions and structures that influence how a risk behaviour is learned, adjusted and then routinised through a process of reflexivity [21, 23]. Research shows that social conditions and structures for sports betting are continuing to evolve through advancements in mobile technology, inducement marketing, and structural changes to betting products [13, 47]. This suggests that young men are also adjusting their knowledge, skill, and control of sports betting through a process of reflexivity, which may have significant implications for their risk for harm. Lessons from public health research for alcohol consumption highlight that public health strategies that target alcohol consumption are therefore unlikely to effectively prevent risk behaviour. [18]. Similarly for young men and sports betting, strategies to address young men’s risk for harm should be reflective of the many domains that may contribute to harm, moving beyond a sole focus on individual responsibility [48].

Fourth, the findings highlight a number of areas for intervention. As argued by public health researchers in obesity prevention, industries that promote personal choice and responsibility in their marketing and promotional messages associated with unhealthy products, must contain accurate and honest information about risk [49]. For gambling, this could mean evidenced-based risk information developed independently from the gambling industry that provides honest and clear information about gambling products, rather than the existing personal responsibility framing that is so dominant in current public education campaigns. These campaigns should focus on the risks associated with gambling, and gambling products. Research indicates that public health messaging through mass media in other areas of public health such as alcohol consumption, smoking, and physical exercise can have a positive effect as part of a comprehensive public health approach to harm prevention [50]. There are a few ways to ensure public health messages can better resonate with target audiences such as young men. For example, creating campaigns that focus on denormalising behaviour using social norm messages [51], as well as ensuring that public health messages are sustained and regularly encountered by target audiences [52]. Moreover, public education campaigns are more effective in preventing and reducing harm when they are implemented alongside upstream strategies of regulatory and policy reform [53]. For example, these upstream initiatives could focus on further regulation in relation to inducement-based marketing. There are some current measures in Australia to prevent sports betting operators from offering inducements relating to sign up promotions [54] and recent inquiries into gambling in the United Kingdom have recommended similar bans for inducements [55, 56]. Lessons from public health show that measures that target the availability and marketing of products, such as alcohol and unhealthy food, are more effective in preventing and reducing harm [57–59]. Given the risks associated with participant’s interactions with inducements and sports betting products, we would argue for a similar approach to policies for gambling to prevent and reduce young men’s risk for harm.

### Limitations

There are some limitations to consider when interpreting the findings. First, it is important to acknowledge that the study was mostly conducted during the COVID-19 pandemic and involved a specific group of young men who were mainly employed in the trades and services industry from Victoria, Australia. While the study provided an in-depth interpretation of the sports betting experiences of young men through a theoretical lens of risk, future research should consider investigating gambling and risk among young men in other settings and who have different characteristics, to broaden knowledge of this public health issue. Second, it is important to note that there may have been some social desirability bias in the responses participants gave during the interview. However, the use of open text responses and prompts to encourage open dialogue, and modifications to strengthen the interview schedule, indicated that it contributed to detailed insights provided by participants and reducing socially desirable biased responses.

## Conclusion

There are a range of socio-cultural and commercial factors that shape young men’s risk perceptions and contribute to their sports betting engagement. This study highlights the importance of broadening public health strategies that focus on individual determinants for gambling to the range of social and commercial determinants. A strong, independent evidenced-based approach is needed to ensure that public health responses to prevent and reduce gambling related harm among young men reflect the diverse meanings and experiences attached to their sports betting engagement.

## Data Availability

The dataset analysed in the current study is not publicly available, or available on reasonable request from the corresponding author because participants explicitly consented to only have their data shared with the immediate research team.

## Abbreviations

PGIS: Problem Gambling Severity Index
RSL: Returned and Services League of Australia
SMA: Social messaging apps
WHO: World Health Organisation

## Footnotes

1 Some Australian and international fantasy sporting competitions require a subscription fee however they are not defined as gambling.
